# Heparanase: A Challenging Cancer Drug Target

**DOI:** 10.3389/fonc.2019.01316

**Published:** 2019-11-28

**Authors:** Deirdre R. Coombe, Neha S. Gandhi

**Affiliations:** ^1^School of Pharmacy and Biomedical Sciences, Curtin Health Innovation Research Institute, Faculty of Health Sciences, Curtin University, Perth, WA, Australia; ^2^School of Mathematical Sciences and Institute of Health and Biomedical Innovation, Faculty of Science and Engineering, Queensland University of Technology, Brisbane, QLD, Australia

**Keywords:** heparanase, heparan sulfate, heparin, drug discovery, cancer, tumor progression, heparanase-2

## Abstract

Heparanase has been viewed as a promising anti-cancer drug target for almost two decades, but no anti-heparanase therapy has yet reached the clinic. This endoglycosidase is highly expressed in a variety of malignancies, and its high expression is associated with greater tumor size, more metastases, and a poor prognosis. It was first described as an enzyme cleaving heparan sulfate chains of proteoglycans located in extracellular matrices and on cell surfaces, but this is not its only function. It is a multi-functional protein with activities that are enzymatic and non-enzymatic and which take place both outside of the cell and intracellularly. Knowledge of the crystal structure of heparanase has assisted the interpretation of earlier structure-function studies as well as in the design of potential anti-heparanase agents. This review re-examines the various functions of heparanase in light of the structural data. The functions of the heparanase variant, T5, and structure and functions of heparanase-2 are also examined as these heparanase related, but non-enzymatic, proteins are likely to influence the *in vivo* efficacy of anti-heparanase drugs. The anti-heparanase drugs currently under development predominately focus on inhibiting the enzymatic activity of heparanase, which, in the absence of inhibitors with high clinical efficacy, prompts a discussion of whether this is the best approach. The diversity of outcomes attributed to heparanase and the difficulties of unequivocally determining which of these are due to its enzymatic activity is also discussed and leads us to the conclusion that heparanase is a valid, but challenging drug target for cancer.

## Introduction

Heparanase is a heparin/heparan sulfate (HS) specific endo-β-D glucuronidase. It was originally called a heparan sulfate degrading endoglycosidase, heparitinase or heparinase, and the human enzyme was first isolated from placenta (1) and later from platelets (2), whereas the mouse enzyme was initially isolated from a murine mastocytoma (3). In the mid-late 1980s, we and others demonstrated that the ability of heparin to inhibit metastasis was consistent with its ability to inhibit heparanase released by tumor cells, and this activity was independent of heparin's anticoagulant activity (4, 5). This finding firmly placed heparanase as cancer-associated enzyme. In 1999 the cloning of heparanase from human platelets, and the discovery that the sequences of heparanase from human activated murine T lymphocytes and rat adenocarcinoma cells were highly homologous, confirmed that the heparanase activity detected in normal mammalian cells and the tumor enzyme are the same (6). Another 1999 study published back-to-back with the above paper, also reported the cloning of human heparanase, and here it was reported the heparanase gene was preferentially expressed in tumor tissue compared to normal tissue (7). They found when poorly metastatic murine melanoma and T-lymphoma cells were transfected with the heparanase gene, this resulted in a massive increase in metastases (7). These papers led to a plethora of studies focusing on the development of heparanase inhibitors as anti-cancer agents. Four of these molecules: PI-88 (muparfostat), PG545 (pixatimod), SST0001 (roneparstat), and M-402 (necuparanib) have progressed to clinical trials. Despite impressive effects in a variety of preclinical models, the clinical data have been less convincing, and development of some of these compounds has discontinued or has stalled (8).

As has been highlighted in recent reviews, it is now known that heparanase is involved in a range of pathologies in addition to cancer, including inflammation, diabetes, bone necrosis, liver fibrosis, amyloidosis and Alzheimer's disease, and in the infection and spread of numerous viruses (9–12). The contributions of this protein to normal physiological and disease processes are complex, particularly given that heparanase has been reported to act in enzymatic and non-enzymatic ways (9, 13, 14). The possibility that heparanase possesses more than one functional site: a catalytic site and at least one other functional site, complicates the interpretation of heparanase gene knock-out studies and may complicate the development of heparanase inhibitors. Although there is no evidence for more than one enzymatically active mammalian heparanase, a second closely related protein called heparanase-2, or Hpa2, was cloned in 2000 (15). In contrast to heparanase, Hpa2 lacks catalytic activity (16). However, like heparanase, it has a role in cancer, but generally in tumor suppression, not tumor promotion as is seen with the catalytic protein (17). In adults, heparanase is also involved in normal physiological processes like tissue regeneration and repair, wound healing, hair growth, dendritic cell migration, and the implantation of embryos during the early stages of pregnancy (18, 19).

While this manuscript was in preparation an extensive review on heparanase in cancer highlighting the numerous synthetic and chemically modified natural compounds that have been produced as potential drugs targeting heparanase was published (20), accordingly we will take a different approach here. In this article, we look at heparanase from the viewpoint of heparanase as a drug target, and ask why has the translation from preclinical studies to successful clinical trials of drug candidates targeting heparanase in cancer been so difficult?

### Heparanase Structure

Heparanase is produced as a preproenzyme; the signal sequence is removed upon entry into the endoplasmic reticulum (ER) giving rise to a 65 kDa latent proenzyme which is secreted. At the cell membrane the proenzyme binds to HS-proteoglycans and of these, the syndecans, are particularly important for complexing with the enzyme, then internalizing and transporting it to the late endosomes or lysosomes where the proenzyme is processed (21). Heparanase can also be internalized by a HS independent mechanism by binding to mannose-6-phosphate receptors on the cell surface (22). Purification of active platelet heparanase revealed the active enzyme is a non-covalently linked heterodimer comprising the N-terminal 8 kDa fragment, and the 50 kDa C-terminal fragment of proheparanase; therefore, processing resulted in the excision of an internal linking segment (23). Sequence alignment and folding prediction studies suggested heparanase adopts a (β/α)_8_-TIM barrel fold (eight alternating β-strands and α-helices), a motif common in glycosidases. In heparanase, both fragments were predicted to contribute to the TIM barrel fold, with a β/α/β element coming from the 8 kDa fragment and the remaining alternating β-strands and α-helices coming from the 50 kDa fragment (24).

**Figure 1:**
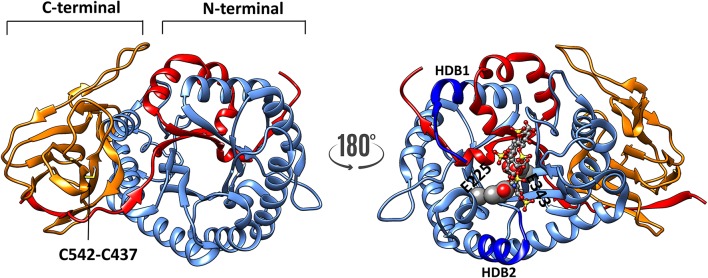
The three-dimensional structure of heparanase (PDB code: 5e9c) showing the 8 kDa unit as red ribbon, whereas the catalytic (TIM barrel) is shown in blue. The C-terminal 413–543 shown in orange ribbon. There is a disulphide bond C437-542 (indicated by an arrow) present in both activated heparanase and proheparanase. The disulphide bond and heparin tetrasaccharide are shown in sticks representation, whereas the catalytic residues are shown as spheres, respectively. HBD1 and HBD2 (dark blue) refer to heparin binding sites consisting of residues 158–171 and 270–280, respectively.

The crystal structure of human heparanase has verified these predictions (25). A baculovirus dual-expression system was used to produce the active enzyme for the crystal structure. The cDNAs encoding either the 8 kDa fragment or the 50 kDa fragment were both placed into a single bacmid, but under the control of different viral promotors, this enabled the two fragments to co-translationally fold into the mature enzyme. The crystal structure also confirmed the (β/α)_8_-TIM barrel fold is flanked by a smaller COOH-terminal β-sandwich domain (C-domain) (Figure 1), which had been described previously from a predicted three-dimensional structure (26). The C-terminal region includes a hydrophobic and conserved sequence (residues 515–534) (27) and a disulfide bond between Cys^437^ and Cys^542^ (Figure 1), which is required for the activation and secretion of heparanase (28). Site-directed mutagenesis confirmed that the catalytic mechanism uses a proton donor (Glu^225^) and a nucleophile (Glu^343^), and consistent with the active sites of other glycosyl hydrolases, these two amino acids are essential for the catalytic activity of heparanase (29). The crystal structure revealed these two residues are in a cleft ~10 Å long located in the (β/α)_8_ domain, and lined with the basic side chains of arginines and lysines (25). The crystal structure of proheparanase confirmed that the 6 kDa linker, which is excised in native, active heparanase, forms a helix which is located immediately above the active site cleft sterically blocking all but a small pocket containing the catalytic residues. Although the linker region sterically blocks the access of bulky HS substrates to the cleft, it may be a binding site for smaller endogenous substrates the nature(s) of which are unknown (30). These structural data indicated that the internalization of proheparanase that occurs though binding to cell surface HS-proteoglycans, must occur via an HS binding site that is distinct from the catalytic cleft. It is unclear as to what region of heparanase is involved at the cell surface in HS-independent internalization. Although specific amino acids within the hydrophobic region of the C-terminal region of heparanase were found to be required for heparanase activation, the data are consistent with a role in heparanase intracellular trafficking and secretion rather than in binding to a cell surface protein (27).

**Figure 2:**
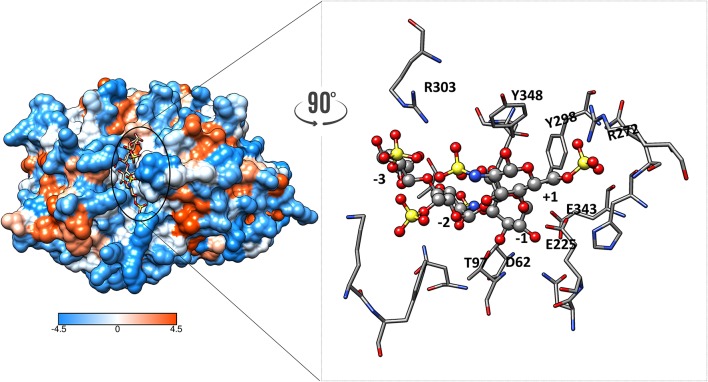
Hydrophobic surface of heparanase showing heparin tetrasaccharide in the catalytic cleft. The orange color indicates hydrophobicity whereas the blue show hydrophilic surfaces. Zoom-in view of the catalytic cleft in complex with the heparin tetrasaccharide substrate showing interactions at various subsites. Hydrogens are not shown for clarity.

The structure of heparanase in complex with HS analogs or with a heparin tetrasaccharide was determined from crystallization studies at pH 5.5, the optimal pH for heparanase enzymatic activity. These data revealed the nature of the bonds that retain the HS substrate in the cleft. Interestingly, these are predominately hydrogen bonds. There is a network of direct hydrogen bonds linking the tetrasaccharide substrate, which is the smallest HS or heparin fragment cleaved, with non-basic amino acids at the base of the cleft (Figure 2). These amino acids are Gly^389^, Asn^64^, Tyr^391^, Gly^349^, Gly^350^, Thr^97^, Asn^224^, Gln^270^, and Asp^62^. In contrast, only three amino acids appear to be involved in electrostatic interactions, and these are Lys^159^, Arg^272^, and Arg^303^ (25, 31). Earlier reports where homology modeling was used to predict the amino acids interacting with the HS substrate suggested electrostatic interactions with basic residues made a greater contribution (32, 33). We have examined heparanase in four different animal species to determine whether the critical amino acids for substrate binding in the human enzyme are conserved. This study revealed that the catalytic residues are conserved in the species examined, as were the amino acids involved in hydrogen bonding except for Asn^64^ and Thr^97^, and of the residues involved in electrostatic interactions only Lys^159^ was not conserved (31). In contrast, the amino acids surrounding the HS binding residues were much less conserved. Thus, it is likely that the positively charged amino acids serve to direct the HS chain into the catalytic cleft, but hydrogen bonding is of key importance for stable substrate binding.

### Heparanase Substrate Recognition

The substrate specificity of heparanase has been extensively investigated. A study using a series of structurally defined oligosaccharides ranging in size from penta- to nona-saccharides where the heparanase cleavage products were examined by electrospray ionization mass spectrometry (ESI-MS), revealed that heparanase most favors cleaving the linkage of a glucuronic acid linked to 6*O*-sulfated glucosamine that may be *N*-sulfated or *N*-acetylated (34, 35). Heparanase was found to be a strict endo-β-glucuronidase, only cleaving internal linkages. In fragments of repeating –GlcUA-GlcNS6S/GlcNAc6S- not every –GlcUA-GlcNS6S- linkage is cleaved, rather consecutive linkages of this structure are cleaved when GlcNAc6S is a component of the non-reducing end, trisaccharide domain, which is not cleaved. However, if this residue is replaced with GlcNS6S, the immediate next cleavage site is skipped to produce a bigger fragment from the middle portion of the substrate (34). Molecular modeling of heparanase co-crystallized with tetrasaccharides from heparin revealed why a GlcUA2S or an IdoUA2S is not part of the cleavage site; steric clashes occur between heparanase Asn^224^ and the bulky 2*O*-sulfate (25). Moreover, when heparin type ligands containing IdoUA bind to heparanase the IdoUA residues are constrained in the ^2^S_0_ conformation and cannot undergo the conformational changes that are required for cleavage (25, 36), and so they act as competitive inhibitors of HS cleavage by heparanase. This study also revealed why the favored trisaccharide for cleavage has an *N*-sulfated glucosamine in the −2 position and 6*O*-sulfated glucosamine in the +1 position. The binding of heparanase introduces a bend across −2, −1, +1 of the trisaccharide, and the bend separates the 2*N* and 6*O* sulfates allowing the catalytic residues of heparanase to more easily access the anomeric center of the −1 GlcUA and so catalyse cleavage (25). In this context, the 2*N* and 6*O* sulfates have been described as “mechanistic handles” that heparanase uses to open the HS helix, as well as acting as a recognition signal to direct the enzyme to particular glycan structures in the HS chain that are permissible for cleavage (25).

## Functions of Heparanase in Cancer Biology

### Extracellular Heparanase Functions Associated With Enzymatic Activity

The enzymatic function of heparanase is important both within the cell and extracellularly. Extracellularly heparanase is one of an array of enzymes that act on the extracellular matrix and basement membrane surrounding a primary tumor to weaken these structures and so facilitate tumor cell invasion into the surrounding tissues and assist metastasis formation. Experimental heparanase overexpression in a transgenic mouse model verified extensive HS fragmentation *in vivo* (37). The fragmentation of HS chains was the first appreciated function of this protein, and it remains the key heparanase function assayed by the majority of drug discovery programs targeting heparanase (5, 20, 38). The degradation of HS chains on matrix proteoglycans by heparanase, as well as dissolving the physical barrier, also releases latent growth factors, like vascular endothelial growth factor (VEGF), basic fibroblast growth factor (FGF2), hepatocyte growth factor (HGF), transforming growth factor-β (TGF-β), and keratinocyte growth factor (FGF4) which are bound and sequestered to the matrix by HS (20). Cleavage of HS gives these signaling molecules better access to their receptors. Indeed, FGF2 signaling is enhanced by moderate heparanase activity possibly because the formation of FGF2-FGFR-HS complexes necessary for sustained signaling is facilitated (39). Also, heparanase overexpression has been shown to produce HS structures that more readily facilitate FGF and FGF receptor complex formation than HS structures obtained when heparanase is not overexpressed. This is probably because 6*O-*sulfotransferase, an enzyme involved in HS and heparin biosynthesis, is upregulated in heparanase overexpressing tissue, resulting in increased 6*O*-sulfation of HS (40). The notion that heparanase, through its enzymatic activity, releases sequestered pro-angiogenic growth factors like VEGF, and so facilitates VEGF receptor interactions and angiogenesis in tumor models has been demonstrated (41). In addition, there are examples where heparanase gene silencing has resulted in tumors that are less vascularised and less metastatic than their controls (42, 43), all of which very strongly point a contribution of heparanase in tumor angiogenesis.

Heparanase degrades HS chains on cell surface proteoglycans as well as on proteoglycans within the extracellular matrix. The cell surface proteoglycan that has received the most attention in this regard is syndecan-1. This proteoglycan potentially has two HS chains and three chondroitin sulfate chains covalently linked to its extracellular domain. The trimming of these HS chains by heparanase exposes a site on the syndecan-1 core protein that is susceptible to cleavage by proteases, causing shedding of the extracellular domain of syndecan-1 from the cell surface (44). The shed syndecan can bind to endothelial cells or tumor cells via the VEGF receptor, VEGFR2, and/or the integrin α4β1, to cross-link these two proteins. This causes the activation of VEGFR2 and the trafficking of the α4β1-VEGFR2 complex to the leading edge of the cell where it facilitates tumor, or endothelial cell, migration via Rac1 (Ras-related C3 botulinum toxin substrate 1) activation and the reorganization of the actin cytoskeleton (45). This mechanism [reviewed in (46)] may explain how heparanase's enzymatic activity can enhance both tumor cell invasion and angiogenesis.

Heparanase (either active or latent) has been reported to cluster syndecans on the cell surface thereby enhancing cell spreading and adhesion. The mechanism for these effects involves Rac1 activation in a process that does not require heparanase enzymatic activity (47). Studies with heparanase, and a disulfide-linked heparanase peptide dimer (Lys^158^-Asp^171^Cys x2), that contains an amino acid sequence that binds heparin/HS (48), were interpreted as demonstrating that heparanase cross-links or clusters cell surface syndecans to facilitate cell adhesion and spreading, processes mediated by PKCα (protein kinase C alpha), Rac1, and Src (a proto-oncogene tyrosine-protein kinase) (49). The peptide dimer was more potent than intact heparanase in these assays, but unlike heparanase-syndecan complexes which were internalized, the peptide dimer-syndecan complexes remained on the cell surface. Data from the crystal structure of heparanase, indicated that the two HS binding sites proposed from the sequence, Lys^158^-Asp^171^ and Gln^270^-Lys^280^ are located at either end of the HS binding cleft (25, 48), and our analysis of the crystal structure indicated that the Gln^270^-Lys^280^ region is of major importance for HS binding in the intact proheparanase and also in heparanase. A third potential HS binding region identified from the heparanase sequence, Lys^411^-Arg^432^ and which includes the motif KRRKLR (48), appears not to be involved in HS binding as in the folded protein this motif is largely buried (25). When expressed as peptides, it was reported that the Lys^158^-Asp^171^ peptide exhibited a higher affinity for heparin/HS than the Gln^270^-Lys^280^ peptide (48), which is in contrast to our findings using the crystal structures of both activated heparanase and proheparanase. Indeed, from our analyses, it appears as if there is a single HS binding region that includes Lys^158^ and Lys^159^ along with the Gln^270^-Lys^280^ region. Thus, it is possible that the peptide dimer may not be a good model for heparanase and hence may not mirror the behavior of heparanase in these assays. Nevertheless, both enzymatic and non-enzymatic functions of heparanase are probably involved in cell invasion and in the pro-angiogenic activity of this enzyme (10, 50), and it is likely that syndecan clusters are involved, but whether heparanase, on its own, clusters syndecan is questionable from these data.

The HS fragments produced as a result of heparanase cleavage can have signaling activities in their own right. For example, heparanase was shown to stimulate the release of pro-inflammatory cytokines [interleukin (IL)-1β, IL-6, IL-8, IL-10, and tumor necrosis factor (TNF)-α] from peripheral blood mononuclear cells with the mechanism being attributed, in part, to the binding of HS fragments, released by heparanase cleavage, to Toll-like receptor-4 (TLR4); thereby stimulating signaling through the MyD88 (myeloid differentiation primary response 88) pathway (51). However, heparanase also appears to influence cytokine secretion from immune cells in the absence of enzymatic activity. Heparanase caused activated T cells to shift from a T-helper (Th)1 profile to a more Th2 profile as indicated by the cytokines released by activated splenic lymphocytes, there being decreased levels of IL-12 and TNF-α and increased levels of IL-4, IL-6 and IL-10 released, an effect that was independent of its enzymatic activity (52). Interestingly, macrophages isolated from heparanase knock-out mice expressed lower levels of various inflammatory cytokines and chemokines, including TNF-α, IL-6, IL-10, IL-1β, and CXCL2 (also called macrophage inflammatory protein 2-alpha) (53). These heparanase negative macrophages also displayed diminished migration and phagocytic capacity compared to wild type (WT)-macrophages, suggesting that heparanase is important for macrophage activation and function. This study also showed that heparanase negative macrophages, in contrast to WT-macrophages, could not invade tumor tissue in response to CXCL2 and did not attenuate tumor growth (53). In another study using gene-modified mice that lacked heparanase in natural killer (NK) cells, it was demonstrated that endogenous NK cell heparanase was required for effective tumor NK cell invasion and immune-surveillance. The data suggested that heparanase deficient NK cells were unable to degrade the tumor extracellular matrix and invade the tumor. As a result, the mice were susceptible to tumor growth following inoculation with cell lines of prostate or mammary carcinoma, metastatic melanoma, or lymphoma (54). These two examples indicate that heparanase can be beneficial for combatting tumor growth, as well as detrimental; the latter being more commonly highlighted. The contribution of heparanase to leukocyte behavior and tumor progression is discussed in detail elsewhere in a recent review (55), where it is made clear that heparanase can enhance tumor clearance by facilitating immune cell infiltration of tumors, as well as promote tumor survival by facilitating tumor cell migration.

### Extracellular Heparanase: Non-enzymatic Activities

Several cell adhesion pathways are regulated, or mediated by heparanase in a manner that does not involve enzymatic activity. For example, heparanase was shown to induce the clustering of breast cancer cells in a manner reminiscent of circulating tumor cells, and knockdown of heparanase inhibited the formation of these cancer cell clusters and suppressed breast cancer metastasis (56). Heparanase has also been shown to augment angiogenesis by a mechanism that involves activation of the β1 integrin/hypoxia-inducible factor (HIF)-2α/fetal liver kinase 1 (Flk-1)/P38 MAP kinase/heat shock protein (HSP)27 adhesion and signaling pathway, a mechanism that does not involve enzymatic activity or the release of cytokines by HS degradation (57). Another non-enzymatic function of heparanase is the activation of Akt (a protein kinase with roles in apoptosis, cell migration, and proliferation), most likely by a mechanism involving cross-talk between a putative heparanase cell surface receptor and integrins (58). Data were reported, which suggested that heparanase stimulation activates PI3-kinase (PI3K) which then phosphorylates Akt. Integrins were found to promote the activation of the PI3K-Akt pathway by heparanase, via a mechanism that involves focal adhesion kinase (FAK) and proline-rich tyrosine kinase 2 (PYK2) auto-phosphorylation. It was also shown that exposure to heparanase increased resistance to stress-induced apoptosis, and this was assumed to involve the PI3K-Akt pathway, but this was not proven (58).

Heparanase has been shown to have a direct effect on the coagulation system via mechanisms that are independent of enzymatic activity. In humans, both the 50 kDa (active) and 65 kDa (latent) forms of heparanase are abundant in platelets and are released following the interaction of thrombin with the protease-activated receptor (PAR)-1 on the platelet surface leading to platelet activation. A similar mechanism was shown to release heparanase from granulocytes (59). Heparanase from these sources forms a complex with tissue factor (TF) which leads to increased factor Xa levels and procoagulant activity. This effect is regulated by tissue factor pathway inhibitor-2 (TFPI-2) binding heparanase and inhibiting the TF/heparanase complex. Heparin also inhibits the interaction between TF and heparanase. The region on heparanase, which was identified from a collection of heparanase peptides as involved in procoagulation and TF binding, spans amino acids 423–438. This region was described as a weak heparin binding domain and is not involved in the catalytic function of heparanase (13, 60). However, this region is largely buried in the intact protein (25), raising the question as to whether, or which part of, the region spanning amino acids 423–438 is involved in TF binding in the intact protein. Interestingly, peptides from TFPI-2 that inhibited the procoagulant activity of heparanase, but not the catalytic activity, also reduced tumor growth and vascularisation in murine tumor models (61). It is likely that platelet heparanase contributes to platelet-endothelial cell adhesion and the hyper-thrombotic conditions seen in cancer patients via another mechanism. This mechanism involves P-selectin, on activated platelets, binding HS on endothelial cells that have been trimmed by heparanase (62). A potential regulator of heparanase released into the circulation from platelets and leukocytes is the relatively abundant plasma protein, histidine rich glycoprotein (HRG). HRG interacts directly with heparanase to enhance its enzymatic activity, and this interaction is inhibited by HS. Heparanase binding to cell surfaces is also partially inhibited by HRG, and it was suggested this had the effect of maintaining heparanase in a soluble extracellular form rather than it being internalized into cell endosomes (63).

### Intracellular Heparanase

Intracellularly heparanase is primarily located in lysosomes or late endosomes where the acidic environment favors enzymatic activity, although some intracellular heparanase was found to reside in autophagosomes. Further evidence from heparanase knockout mice indicated heparanase might modulate autophagy (64), suggesting it has a role in the normal physiology of lysosomes. In the late endosomes, heparanase has been shown to trim the HS chains of syndecans (65), whilst in mast cells, heparanase is sorted to the secretory granules where it cleaves heparin chains covalently attached to serglycin (66). The HS or heparin fragments produced by heparanase cleavage range in size from 5 to 20 kDa indicating heparanase regulates the size of the free HS/heparin chains secreted from mast cells rather than causing the total degradation of HS/heparin chains.

Heparanase activity in the late endosomes has an outcome other than the fragmentation of HS chains. It regulates exosome excretion and the composition of proteins within secreted vesicles so that cells with high levels of heparanase release more exosomes and these vesicles contain higher levels of HS binding growth factors like VEGF and hepatocyte growth factor (HGF) as well as syndecan-1 (67). Moreover, the effects of heparanase on exosome secretion were dependent on HS cleavage, and the production in endosomes of syndecans with trimmed HS chains that can undergo proteolytic cleavage to generate syndecan C-terminal fragments that remain within the membrane, and cluster into microdomains. By this means, heparanase is a key modulator of exosome biogenesis that occurs by the syndecan-syntenin-ALIX (a regulator of the endo-lysosomal system) pathway (65, 68). An issue that remains unresolved is to what extent the cargo contained in these exosomes is responsible for the effects attributed to heparanase in sustaining tumor growth, tumor cell invasion, and the augmentation of angiogenesis, rather than heparanase itself. Most interestingly, the exposure of myeloma cells to various chemotherapy drugs dramatically enhanced the secretion of exosomes containing high levels of the 65 kDa form of heparanase as a cargo (69). The interaction of macrophages with these exosomes caused enhanced macrophage migration and secretion of TNF-α, a myeloma growth promoting cytokine. The latent heparanase was located on the surface of these exosomes and was readily taken up by tumor cells and activated, giving it the ability to modify the tumor microenvironment and so facilitate disease progression (69).

A significant fraction of heparanase is located in the nucleus. In glioma and breast carcinoma, this was found to be about 7% of the cytosolic enzyme, and nuclear heparanase was enzymatically active, degrading nuclear HS (70). Indeed, heparanase was found to co-localize with syndecan-1 in the nucleus of mesenchymal tumors (71). Curiously, given the association of heparanase with cancer, the translocation of heparanase into the nucleus has been associated with cell differentiation; the differentiation of HL-60 cells into monocytes and macrophages (72), and the differentiation of, and expression of differentiation markers by esophageal keratinocytes (73) being two examples. Possibly the intracellular location of heparanase may be a prognostic indicator for some cancers. In squamous cell carcinoma of the head and neck, nuclear localization of heparanase predicted a good outcome, whereas cytoplasmic heparanase correlated with a poor prognosis (74). More specifically, nucleolar and nuclear heparanase were proposed to have contrasting effects on cell proliferation. Nuclear heparanase was reported to be involved in differentiation, but active nucleolar heparanase was said to augment cell proliferation via its modulation of DNA topoisomerase I activity, an enzyme essential for DNA replication and gene transcription that is inactivated by HS (74). Other studies suggested that heparanase can bind DNA and/or chromatin. One study demonstrated, using atomic force microscopy, that heparanase bound plasmid DNA, most probably by a charge mediated interaction (75). Another study found evidence that heparanase (likely the ~50 kDa form) belongs to a group of chromatin-associated proteins that can modulate gene transcription. More specifically, using T lymphocytes, heparanase was found to preferentially associate with euchromatin. It was associated with the promoters and 5′ coding regions of a large number of different genes and was part of an active transcription complex necessary for the transcription of a subset of inducible immune response genes. The evidence further suggested that heparanase acts by binding LSD1, a lysine specific demethylase, thereby preventing recruitment of MLL, a histone methylase, resulting in modification of histone H3 methylation patterns to one associated with inducible gene transcription (76). Such effects are due to the binding capability of heparanase rather than its catalytic activity.

If heparanase has the potential to alter gene transcription either by directly binding DNA or indirectly by regulating methylation patterns, cancer cells with high levels of endogenous heparanase should express a different pattern of genes than cells with low or normal heparanase levels. This was found to be the case in melanoma cells subjected to siRNA mediated knock-down of heparanase expression. The genes down-regulated by heparanase silencing were all categorized as nucleosome genes or nucleosome assembly genes, whereas numerous pro-apoptotic genes were up-regulated following heparanase silencing. Moreover, a significant increase in apoptosis was detected in the absence of heparanase that involved the caspase 3/poly(ADP-ribose) polymerase-1 (PARP-1) pathway, as revealed by the appearance of fragmented caspase 3 and PARP-1 (77). This study suggested that heparanase may directly interfere with apoptotic pathways, and so could protect cancer cells from apoptosis during therapy. Clearly, more work is required to unravel the complexity of heparanase's activities in the nucleus.

## Splice Variants of Heparanase

In addition to full-length heparanase alternatively spliced variants of human heparanase have been detected. A splice variant lacking exon 5 was detected in cDNA from a renal cell carcinoma infiltrated kidney. The reading frame of the wild-type gene was conserved in this variant, resulting in a protein lacking 58 amino acids, including the active site proton donor Glu^225^ (78). Very little is known about this variant; but from the reported study, it did not appear to be secreted, it is not cleaved, and it lacks enzymatic activity. Another variant termed T5, has attracted more attention. This truncated, enzymatically inactive heparanase variant arises when 144 base pairs of intron 5 are joined with exon 4, giving rise to a protein of between 15 and 17 kDa, depending on its glycosylation status (79). The T5 protein does not bind heparin (79), probably because it contains only a few of the amino acids identified from the crystal structure of heparanase that are involved in binding heparin (25). In addition, its structure may not allow heparin binding because it is likely T5 only partially resembles active full-length heparanase in its three-dimensional conformation. This conclusion is supported by monoclonal antibody (mAb) data which suggests that in T5 the linker region is exposed on the outside of the protein and is strongly immunogenic (80), whereas the linker region is excised in active heparanase.

Like heparanase, T5 significantly enhanced tumor development in a myeloma xenograft model, despite its inability to degrade HS (79). Interestingly, the effect of both T5 and heparanase in this model was not apparent until 3 weeks post subcutaneous inoculation when the development of tumor xenografts expressing either T5 or heparanase was markedly enhanced compared to controls lacking these proteins. Both the density of blood vessels and vessel maturation were enhanced with T5 or heparanase expression, but T5 expression better-allowed pericyte coverage of blood vessels and small capillaries (79). *In vitro* studies indicated that expression of these proteins increased the proliferation of myeloma cells and enhanced colony formation in soft agar of myeloma, pharynx carcinoma, and human embryonic kidney cells. Human embryonic kidney cells and myeloma cells overexpressing T5, or heparanase, displayed enhanced Src phosphorylation, whereas Erk (extracellular signal regulated kinase) phosphorylation was not affected, and in the case of T5 this effect was independent of HS. The use of Src inhibitors caused colony formation in T5 and heparanase expressing cells to resemble that of control cells, which led the authors to conclude that Src activation contributes to the enhanced cell proliferation seen with T5 or heparanase expression. Finally, a cohort of renal cell carcinoma specimens was examined for both T5 and heparanase mRNA and were immunostained with a mAb that preferentially recognizes T5 over heparanase. These data indicated that the intensity of T5 staining was positively associated with tumor size and grade, and when T5 mRNA was detected so to was heparanase mRNA (79, 80). A very recent study using glioma revealed that T5, like heparanase, was associated with the upregulation of CD24, a protein that is significantly associated with malignancy and poor outcomes in a number of carcinomas (81). Thus, the T5 variant of heparanase displays many of the biological effects of the full-length protein, at least in the cancer models studied to date, yet it lacks enzymatic activity.

## Heparanase-2 (Hpa2)

### Structure and Biochemistry

The publication of the sequence of heparanase prompted two groups to use this sequence to search EST databases to determine if mammalian heparanase was one of a family of enzymes. From this work another heparanase, Hpa2 was identified and cloned (15, 19). Both groups found that alternative splicing produced different variants of Hpa2; McKenzie et al. reported three variants (15), whereas Vreys and David as well as finding the earlier variants, reported a fourth splice variant, hep-2B (19). Of these variants, hep2c (corresponding to hep-2AB from Vreys and David) and hep-2B are secreted, and both have been reported to bind to HS-proteoglycans on cell surfaces (16, 19). However, it is the former of these variants, hep2c/hep-2AB, which has become synonymous with Hpa2 and hence in our discussion, we will be referring to hep2c/hep-AB.

Not surprisingly given the means by which Hpa2 was identified there is a high degree of similarity between heparanase and Hpa2 with the overall identity by sequence alignment being 42–44%, including conservation of the two catalytic residues of heparanase, Glu^225^ and Glu^343^ (16, 19). The homology between heparanase and Hpa2 is most apparent in the 8 kDa N-terminal, and the 50 kDa C-terminal portions of heparanase that result from cathepsin L processing. The linker region that is excised in heparanase is far less conserved (15, 19). Importantly, the amino acid site (Gln^157^-Lys^158^) that is first cleaved by cathepsin L, including the essential Tyr^156^, is not conserved in Hpa2 and is replaced by Phe^194^, Ser^195^, Asn^196^ (19). Moreover, there is no biochemical evidence for similar post-translational processing of Hpa2 as is seen with heparanase (16, 19), which is consistent with its lack of catalytic activity.

**Figure 3:**
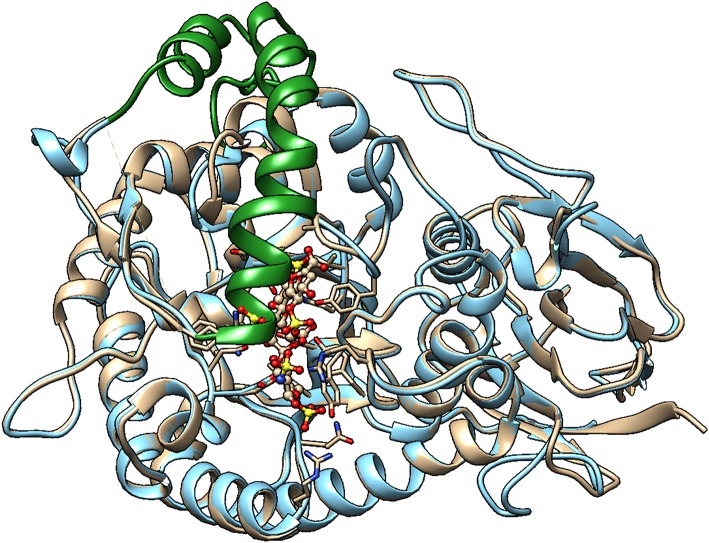
Homology model of Hpa2 was built using the SWISS-MODEL server. PDB 5LA4 (Proheparanase) was used as a template. The obtained homology model of Hpa2 was then superimposed onto active heparanase (PDB code: 5e9c) cocrystal with heparin tetrasaccharide substrate. The linker (green ribbon) of Hpa2 partially occupies the heparin binding site. This is also true for proheparanase (PDB code:5LA4 structure). Heparanase is shown in golden, and Hpa2 in blue.

**Figure 4:**
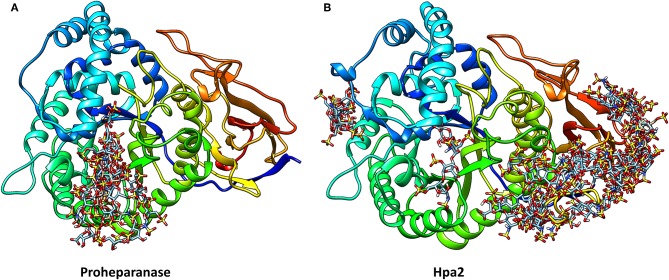
The structures of proheparanase **(A)** and Hpa2 **(B)** are shown in ribbon representation and colored from N- (blue) to C- terminal (red). Prediction of heparin tetrasaccharide binding site using the ClusPro server suggests that most of the tetrasaccharides interact with residues 270–280 in proheparanase, whereas most of the interactions with the tetrasaccharides are concentrated toward the C-terminal domain of Hpa2.

Unfortunately, the published molecular model of Hpa2 is of the region corresponding to the 50 kDa fragment of heparanase, it, therefore, lacks the linker region and the *N*-terminal region (82). Also, it predates the crystal structure of human heparanase (25). We have developed a homology model of Hpa2 using the SWISS-MODEL server and proheparanase as the template [PDB 5LA4 (30)]. In this model, the linker domain in Hpa2 partially occupies the catalytic cleft (Figure 3) and is likely to obscure at least some of the basic residues surrounding the cleft. Of the basic residues lining the substrate binding cleft in heparanase, only Arg^272^ and Arg^303^ are strictly conserved in Hpa2; Arg^273^ is replaced with lysine and His^230^-Lys^231^ becomes a lysine and a serine, but Lys^158^, Lys^159^, and Lys^161^ are not conserved. Similarly, a number of the residues involved in hydrogen bonding with the HS substrate in heparanase are also not conserved. Collectively these findings suggest that if this cleft is available for HS binding, the affinity of the interaction would be lower than that seen with heparanase. However, as Hpa2 has a higher affinity for heparin and cell surface HS than heparanase (16), and given the likely position of the linker domain, the site(s) where heparin/HS binds is not the substrate binding cleft of heparanase. A mAb targeting the heparin/HS binding site on Hpa2 has been reported based on the fact that it inhibits Hpa2 binding to cell surfaces (83). As Hpa2 co-localizes with syndecan-1 on cell surfaces and cell surface binding is inhibited by heparin (16), this is a reasonable conclusion. However, the epitope on Hpa2 recognized by this mAb is not known. The structural similarity of the HS binding site on Hpa2 to the HS binding site on proheparanase, used for cell internalization via cell surface syndecans, is also unknown. To obtain a better understanding of this issue, we subjected both proheparanase and Hpa2 (modeled as shown in Figure 3) to an analysis of HS binding sites using the ClusPro server. This revealed that the most favored region for binding heparin tetrasaccharides is the C-domain of Hpa2, in contrast, for proheparanase it is the region around the heparin binding domain-2 (HBD2) (Figure 4). Thus, it is likely that Hpa2 binds to cell surface HS via its C-domain. An examination of the sequence alignment of these two proteins indicates that Hpa2 has what appears to be an extended heparin binding region in its C-domain spanning amino acids Lys^449^ to Arg^480^. This covers the third potential HS binding region in heparanase, Lys^411^-Arg^432^, and includes the motif KRRKLR (48), which in heparanase, is not involved in HS binding as it is largely buried. In Hpa2 the motif region is: ^465^QR*KPRPGRVIRD*KLR^479^ (the inserted sequence present only in Hpa2 is in italics) and although much of it is buried Arg^466^ is exposed. The analysis shown in Figure 4 indicates that Arg^466^ acts in conjunction with the basic residues in the Hpa2 sequences, His^505^-Lys^512^ and Arg^561^-Thr^568^, to form the HS binding region (Figure 4). Thus, the residues indicated by the ClusPro docking analysis to be involved in binding heparin tetrasaccharides are: Gln^524^, Arg^466^, Lys^509^, Arg^508^, Lys^510^, Lys^512^, Arg^561^, Arg^564^, Arg^567^, and Thr^568^. From the sequence alignment of heparanase and Hpa2, it is apparent that heparanase lacks three of these basic residues, three are conserved and for two residues the charge is retained, but arginine is replaced with a lysine and vice versa. These differences appear to be sufficient to stop heparin from binding to this region in heparanase (Figure 4).

The high affinity of Hpa2 for HS on cell surfaces has direct consequences for heparanase processing and activity. Hpa2 inhibits the internalization of heparanase and hence the processing and activation of this enzyme. It also markedly inhibits the enzymatic activity of purified, cell-free, active heparanase (16). Both of these effects can be attributed to competition for HS binding. However, co-immunoprecipitation data indicated that heparanase and Hpa2 could physically associate (16) and given a physical association is possible, there are two additional ways the enzymatic activity of heparanase may be inhibited by Hpa2. Firstly, the direct binding of Hpa2 to active heparanase may block enzymatic activity, and secondly, because Hpa2 is retained on cell surfaces, it may bind and retain latent heparanase in this location thereby preventing heparanase internalization and activation. It is also possible that the physical association of Hpa2 with heparanase may alter some of the non-enzymatic functions of heparanase. Curiously, Hpa2 expression does not always inhibit heparanase activity, as heparanase activity remained unchanged in pharyngeal carcinoma cells and in bladder carcinoma cells engineered to overexpress Hpa2 (83, 84).

### Hpa2 and Cancer Biology

The expression of Hpa2 was first reported to be upregulated in colorectal cancer compared to normal tissues (85) and there are a number of studies where the expression of Hpa2 was found to be inversely correlated with disease severity. This was initially reported for head and neck carcinoma where there was an inverse correlation between Hpa2 staining intensity and tumor cell dissemination to lymph nodes, and an association between Hap2 expression and prolonged follow-up or disease recurrence (16). Similarly, in bladder carcinoma, an inverse correlation between Hpa2 staining and tumor grade was reported: weak or no Hpa2 staining was found in stage III tumors, whereas stage I tumors stained strongly (84). Whilst normal tissue adjacent to the head and neck carcinoma lesions did not stain for Hpa2, this was not the case for normal bladder epithelium, which stained strongly (16, 84). In the majority (68%) of melanoma metastasis samples, Hpa2 staining was detected, but this dropped to very low levels for brain metastases (86). Xenograft studies using pharyngeal carcinoma cells and bladder carcinoma cells overexpressing Hpa2 revealed Hpa2 expression attenuated tumor growth and was associated with the appearance of differentiation markers, increased collagen deposition and the induction of lysyl oxidase (83, 84). Reduced cell proliferation and reduced blood vessel densities were also detected in the pharyngeal carcinoma model as was a reduction in the expression of Id1, a proangiogenic transcription factor, which induces the expression of VEGF isoforms (83). Similar data were obtained in a pancreatic carcinoma model leading to the interpretation that Hpa2 functions as a tumor suppressor in these carcinomas in a heparanase and HS independent manner (17).

## Heparanase as a Drug Target

### Criteria for a Good Target

It is clear that heparanase has a profound role in the pathophysiology of a variety of different types of cancers; its increased expression is associated with greater tumor size, more angiogenesis, greater metastatic tendencies, and poor prognosis. In addition, as its genetic knockdown in experimental disease models significantly curtails tumor progression (41, 42), it can be concluded that heparanase is a valid cancer drug target. But is it a challenging drug target?

Frequently successful therapeutic targets are enzymes (87), and heparanase is an enzyme. Its substrate specificity is restricted to particular HS or heparin structures, and as revealed by its crystal structure, its active site is a cleft that has strict structural requirements for access and stable binding (25). From a structure-based drug discovery viewpoint this is a good thing. Importantly, crystal structures are now available for both the active enzyme and its latent precursor (25, 30). This means that it is possible to accurately predict where on heparanase a potential drug is binding. This information can then be used to inform the medicinal chemistry, thereby leading to the rational design of compounds that better modulate heparanase activity. For example, the crystal structure could be used to design a compound that fits into the catalytic cleft and so specifically blocks enzymatic activity. A further criterion for a good drug target is its “assayability” (87). For heparanase, the development of good, reliable assays to monitor its enzymatic activity in a manner that is appropriate for high throughput screening has been problematical, with many of the assays being complex and labor intensive. Nevertheless, the development of simple, synthetic substrates with a single point of cleavage that will allow detailed kinetic analyses to be performed may well-lead to the standardization of heparanase assays (88).

Good drug targets are generally not expressed uniformly throughout the body, and their modulation in normal, non-disease situations should be markedly less critical than in the disease (87). Such is the concentration of recent literature on the contribution of heparanase to a variety of different diseases that the reader would be forgiven for concluding that heparanase contributes minimally, if at all, to normal physiological processes. In normal tissues, heparanase expression is restricted to the placenta, lymphoid organs, leukocytes, platelets, keratinocytes, and endothelial cells and mice deficient in heparanase expression lack gross anatomical abnormalities (18). However, heparanase knock-out mice are not without a phenotype. For example, heparanase has a profound role in wound healing. It is normally found in skin and wound granulation tissue, where it stimulates keratinocyte migration and epithelialisation during healing, as well as angiogenesis and blood vessel maturation (89). Curiously heparanase was found to regulate hair growth, hair homeostasis and the differentiation of the inner root sheath in hair follicles (90). In the bone marrow, heparanase modification of the microenvironment was shown to regulate the retention and proliferation of progenitor cells (91). Bone marrow mesenchymal stem cells are weak expressers of heparanase and loss of heparanase activity reduced their self-renewal and proliferation (92). Collectively these data suggest that heparanase has a role in normal stem cell biology. Heparanase also has a role in normal developmental processes like mammary gland development, where its enzymatic activity is required for mammary epithelial invasion and branching (93). Leukocyte heparanase is necessary for immune cell migration into tissues. This was found to be the case for dendritic cells, NK cells, monocytes and macrophages (18, 53, 54, 94, 95). Moreover, heparanase expression is required for the activation of macrophages, and in mast cells, heparanase is an important regulator of protease storage in mast cell granules (53, 96). Given these findings neutralizing heparanase systemically may alter a patient's ability to mount an efficient immune response to an infection, or to a tumor, and to heal wounds in a timely manner. However, it is recognized that knocking out a gene and neutralizing activity via administering a drug are quite different things, as in the latter, drug penetrance will not be 100% and this may vary according to the route of administration. Given that minor side-effects can be tolerated in an anti-cancer drug, it is fair to say that heparanase scores quite positively on this point.

## Challenges for Heparanase as a Drug Target

Heparanase is a multi-functional protein with different parts of the protein being involved in differing aspects of its biological activity. It should be clear from the above discussion that both the enzymatic and non-enzymatic activities of heparanase are likely to contribute to the role this protein plays in cancer pathology. Although the catalytic cleft is involved in the enzymatic activity, it is not clear which regions of heparanase are involved in its non-enzymatic activity. The fact that the T5 splice variant of heparanase, which lacks enzymatic activity and heparin binding capability, also enhances tumor growth and is associated with a poor prognosis raises the question as to the relevance of the enzymatic activity of heparanase in cancer biology. This question is not clarified by the genetic heparanase silencing experiments because this methodology would knock-down both T5 and full-length heparanase. Given the data on the T5 variant, the question must be asked as to whether the enzymatic activity is the critical function of this protein that causes its pro-tumor and pro-metastasis effects.

It could be argued that the good pre-clinical data of the various heparanase inhibitors, selected based on their ability to block enzymatic activity, indicates the importance of HS degradation in tumor biology. However, as the best-studied inhibitors have pleiotropic effects that are not confined to inhibiting the enzymatic activity of heparanase, this argument may not be valid. For example, PG545 was selected from a series of similar structures because of its ability to bind the angiogenic growth factors FGF1, FGF2, and VEGF (presumably via its sulfated saccharide moiety) as well as for its inhibition of the enzymatic activity of heparanase (97, 98). Moreover, recent studies have indicated that the mode of action of PG545 is likely to be complex. Curiously PG545 has been found to elicit autophagy and persistent ER stress in lymphoma cells, which triggered their apoptosis, and this occurred independently of heparanase (99). Whereas, in glioma, PG545 attenuated heparanase augmented autophagy, and this reduced autophagy was associated with decreased cell growth and decreased tumor load (64). PG545 has also been reported to have immunomodulatory effects that are distinct from heparanase, and its stimulation of dendritic cells to produce IL-12 via a TLR9-dependent pathway which then activates NK cells, is now regarded as a key part of the anti-tumor effect of PG545 (100, 101). Similarly, M-402 was designed to inhibit VEGF, FGF2, stromal cell-derived factor (SDF)-1α, P-selectin as well as heparanase (102), and SST0001 inhibits the activation of a number of receptor tyrosine kinases, in addition to its effects on heparanase (103). A recent publication confirmed M-402 was a broad, multi-targeting drug *in vivo* and one of its very interesting effects relevant to pancreatic cancer invasion/metastasis was on the levels of matrix metalloproteinase-1 (MMP-1) and tissue inhibitor of metalloproteinase-3 (TIMP3); it reduced the former and increased the latter (104). Thus, using one or more, of these well-studied inhibitors to confirm that the biological activity under study is due to the enzymatic activity of heparanase is problematical.

To be more certain of targeting heparanase's enzymatic activity has required the development of blocking anti-heparanase mAbs (105). Two mAbs that block enzymatic activity have been reported. One mAb (9E8) was raised using the heparanase peptide Lys^158^-Asp^171^ as the antigen, whilst the other (H1023) used intact active heparanase as the antigen and the hybridomas produced were screened for heparanase binding and inhibition of enzymatic activity (105). Both mAbs inhibited lymphoma growth in xenogeneic murine models via a mechanism that did not involve a direct cytotoxic effect on the tumor cells, but rather seemed to be a result of neutralization of heparanase activity in the tumor microenvironment. Interestingly, these mAbs also inhibited spontaneous metastasis arising from murine ESb T-lymphoma cells. Tumor growth and angiogenesis were further constrained when both mAbs were administered together implying that greater efficacy is achieved when two different epitopes on heparanase are targeted (105). The peptide antigen is equivalent to HBD1. Given that Lys^158^ and Lys^159^ are available for HS binding in proheparanase, and that the mAb 9E8 inhibited proheparanase uptake by cells as well as heparanase enzymatic activity, it is probable the epitope recognized by this antibody is at the extreme N-terminus of this peptide. Although a three dimensional epitope in the region of the catalytic cleft appears likely for H1023, it is still conceivable that H1023 binding to a site on the β-sandwich C-domain may allosterically influence access to the catalytic cleft. Regardless, these mAbs are recognizing different epitopes. Given these data, it appears at first glance that inhibition of proheparanase uptake and heparanase enzymatic activity by mAbs is sufficient for significant anti-lymphoma activity. However, the extent to which non-enzymatic activities attributed to heparanase are also inhibited by these mAbs is not known. In this context it is important to recognize that an intact antibody molecule is approximate twice the mass of heparanase, thus although the heparanase epitope recognized may comprise a few amino acids the area on heparanase masked by antibody binding will be much greater. Interestingly, the efficacy of PG545 in inhibiting tumor growth appears to be superior to these mAbs on weight of drug administered basis, in the dosing regimen used (105). Although, how much of the activity of PG545 is due to its interactions with heparanase and how much to its other activities is impossible to say.

The fact that heparanase is such a multi-functional protein makes it a challenging drug target. All of the heparanase inhibitors under development include in their biological assay portfolio an assay for this protein's enzymatic activity, but other aspects of heparanase's activities that cannot be attributed to its enzymatic activity are generally not well-studied. Nor is it entirely clear which regions of the protein are involved in these non-enzymatic activities.

Evidence has been presented to suggest that the COOH-terminal domain (C-domain) mediates some of the non-enzymatic functions of heparanase, including Akt phosphorylation and cell proliferation, and it facilitates tumor progression in a glioma xenograft model (26). The C-domain, comprising amino acids 413–543, forms a β-sandwich domain comprising eight β-strands arranged in two sheets, with the 8 kDa N-terminal fragment contributing one of the β-strands (25, 26). This domain structure is stabilized by both hydrophobic interactions and a disulfide bond linking Cys^437^ to Cys^542^ (28) (Figure 1). Although not directly involved in the formation of the catalytic cleft, the C-domain is required for enzymatically active heparanase and heparanase secretion. Deletion of the COOH-terminal amino acids Phe^527^-Ile^543^ was found to cause loss of enzymatic activity (26), and more particularly, mutation of Leu^522^, Leu^524^, Phe^531^, Phe^532^, Val^533^, or Ile^534^ within a conserved hydrophobic region in the C-domain also abolished activation of heparanase (27).

Much of the initial work examining functional activities of the C-domain used a construct that lacked the β-strand contributed by Gln^36^-Ser^55^ from the 8 kDa fragment. Although when expressed, this C-domain localized to the Golgi apparatus and stained with a polyclonal anti-heparanase antibody, both features suggestive of a correctly folded protein, its secretion was modest (26). This raised a query as to the stability of this domain in the absence of the β-strand from the 8 kDa *N*-terminal fragment. Accordingly, the expression of a construct comprising Gln^36^-Ser^55^ linked to the C-domain sequence was examined. This protein, termed 8C, was secreted at levels comparable to native heparanase, it markedly induced Akt phosphorylation, and triggered endothelial cells to form tube-like structures in accordance with the proangiogenic activity of intact heparanase. Unfortunately, glioma progression was not studied with the 8C protein (26). When the regulatory elements of the mouse mammary tumor virus (MMTV-LTR) were used to direct expression of the 8C protein to the mammary epithelium of mice, over time (6–10 months) these animals (MMTV-8C mice) spontaneously developed tumors in their mammary and salivary glands (106). These invasive carcinomas were highly proliferative and phosphorylated signaling proteins, STAT3 (signal transducer and activator of transcription), Erk and Akt, were detected in the tumor tissue. In contrast, heparanase transgenic mice did not spontaneously develop tumors. Curiously, the signaling molecules that were phosphorylated in non-transformed cells were not identical for MMTV-8C and MMTV-heparanase mice (mice in which wild-type heparanase expression is regulated by MMTV-LTR) (106). Clearly more work is required to determine whether the 8C protein or the C-domain as expressed in these studies are good models for understanding the function of the native C-domain in wild-type heparanase.

A further complication for heparanase as a drug target is that it is present in extracellular locations and intracellularly, including within the nucleus. The nature of many heparanase inhibitors, that is their size and charge, is such that they cannot gain access into the cell unless they are transported by ligands into the endosomal pathway. The heparin mimetic-type heparanase inhibitors currently in clinical trial will inhibit heparanase internalization mediated by cell surface HS-proteoglycans, and as the internalization of latent heparanase is required for processing it may be thought that these inhibitors will also halt processing. However, these drugs are unlikely to inhibit heparanase uptake that is independent of cell surface HS. Importantly, heparanase binding with high affinity to cation independent mannose 6-phosphate receptors [CIMPR, also called CD222 and insulin-like growth factor 2 receptor (IGF2R)] leads to its internalization in a way that is independent of HS and is not inhibited by heparin (107). Given that CIMPR is ubiquitously expressed and functions in the recycling of growth factors and other ligands it is probable that significant quantities of heparanase are internalized by this mechanism, and particularly so in cells like leukocytes or lymphoma cells that have relatively low levels of surface HS. In the presence of anti-heparanase drugs, this non-HS dependent mechanism is likely to dominate. It could be argued that heparanase located in endosomal pathways is “protected” from the action of the anti-heparanase drugs currently in a clinical trial. This argument also holds for heparanase in the nucleus. Hence, even in the presence of the drug, nuclear heparanase would continue to protect cancer cells from apoptosis by its ability to down-regulate the transcription of pro-apoptotic genes (77), a function that does not involve enzymatic activity.

Despite the structural similarities between heparanase and Hpa2, very little attention has been paid to the latter protein in relation to whether or not it binds to, or sequesters, anti-heparanase drugs, and so down-modulates their activity. Although in many instances, Hpa2 expression is inversely correlated with cancer progression, this is not always the case. For example, in differentiated thyroid carcinoma, both heparanase and Hpa2 are over expressed relative to benign lesions, but Hpa2 expression is extremely elevated, and this expression is confined to neoplastic cells (108). Similarly, in squamous cervical cancer, a progressive increase in Hpa2 expression according to the severity of the lesion was recorded (109). It is known that Hpa2 can down-modulate heparanase processing and enzymatic activity by competing for HS, but does this remain the case in the presence of anti-heparanase heparin mimetic drugs? To our knowledge, this question has not been examined. Given the structures of the Glycol-split heparin mimetic drugs, SST0001 and M-402, it is very likely these drugs would bind Hpa2 at least as well as, if not better than, they bind heparanase. If binding does occur this could decrease the ability of Hpa2 to inhibit heparanase activity.

## Structural Aspects of Heparanase Inhibitors

Prior to publication of the crystal structure of active heparanase in 2015 the majority of non-antibody heparanase inhibitors were either modified natural heparin molecules (e.g., M-402 and SST0001), sulfated oligosaccharides derived from marine organisms (e.g., carrageenans), semisynthetic compounds comprising oligosaccharide backbones that were chemically sulfated (e.g., PI-88, JG3, and PS3), or as is the case for PG545 a sulfated saccharide component covalently functionalised with cholestanyl aglycone (20, 110). The goal was to mimic heparin's structure but remove its anticoagulant activity. Although heparanase can cleave heparin, the majority of the heparin chains released from mast cells lack the cleavage site and therefore act as non-cleavable inhibitors. The heparanase crystal structure indicated that these heparin mimicking inhibitors would not bind in the catalytic cleft, but rather were binding to the heparanase surface in a manner that masked the cleft thereby preventing access to HS chains with cleavage sites. Studies of PG545 binding kinetics provided clues as to its mechanism of heparanase inhibition. It was found to bind heparanase with parabolic kinetics indicating that two sites on heparanase are involved in PG545's binding and inhibition (111). The identification by homology modeling of a hydrophobic channel on heparanase surrounding the catalytic resides (33) suggested a mechanism. It was proposed that the sulfated saccharide moiety in PG545 binds to the positively charged amino acids of the HS binding regions whilst the cholestanol aglycone component occupies the hydrophobic channel (or cleft) thereby obscuring the catalytic site. It was further proposed that this cholestanol, stabilized on heparanase through its binding in the hydrophobic channel, then serves as a binding site for the cholestanol group of another PG545 molecule (111). The result being that PG545 is a more potent inhibitor of heparanase enzymatic activity than similar compounds that lack the cholestanol aglycone (97, 111).

Recently dendrimer glycomimetics that are heparanase enzymatic activity inhibitors were developed (112). The rationale being that the avidity of weak interactions, of the type mediated by sugar fragments binding a protein, is significantly enhanced if multiple copies of the sugar units are displayed on a chemically defined scaffold. The most potent of these dendrimer glycomimetics, with a potency almost comparable to PG545, comprised individual sulfated maltose units linked to form a tetrameric cluster, such that the terminal groups of the dendrimer arms were the sulfated disaccharides. Given the similarity in the basic structure of the saccharide components of PG545 and the dendrimer, it is possible that the latter compound also binds to the positively charged amino acids surrounding the catalytic cleft, rather than in the cleft itself. However, no modeling or structural data were provided to verify this suggestion. This compound was shown to inhibit tumor growth and metastasis in a mouse xenograft model with human myeloma cells (112), but no other activities described for heparanase were examined. Glycopolymers that inhibit heparanase enzymatic activity have also been prepared. A set of glycopolymers comprised a poly-2-aminoethyl methacrylate (PAMA) backbone to which heparin disaccharides, derived by extensive digestion of heparin with heparinase I, II and III, were covalently coupled, and then any unreacted amines were sulfated (113). These compounds were heterogeneous as specific disaccharides structures were not selected prior to coupling. As well as inhibiting heparanase enzymatic activity a selected glycopolymer was further tested in *in vitro* invasion and migration assays using the murine B16 melanoma line; activities which may be attributed to heparanase. The dendrimers and the glycopolymers were prepared without reference to the structure of the catalytic cleft, or to the specificity of the bond that is cleaved in HS, rather they were primarily designed as heparin mimetics.

In contrast, others have used knowledge of the HS bond that is cleaved by heparanase to inform their choice of the disaccharide unit used as the active entity in their anti-heparanase glycopolymers. In particular, as GlcAβ(1,4)GlcNS(6S) is cleavable, GlcNS(6S)α(1,4)GlcA was chosen because it is not cleaved, yet is a structure that fits and binds within the catalytic cleft of heparanase (114, 115). Thus, a glycopolymer, demonstrated to possess comparable anti-metastatic activity to SST0001, and comparable inhibition of heparanase enzymatic activity as heparin at 10 μg/ml, comprised 12 repeating units of pendent GlcNS(6S)α(1,4)GlcA saccharides (115). However, the inhibition of other heparanase activities that do not require enzymatic activity was not addressed. This glycopolymer lacked anticoagulant activity and bound poorly to the angiogenic growth factors, FGF1, FGF2, and VEGF. It also bound poorly to platelet factor 4 (PF4), suggesting it may not trigger thrombocytopenia as was the case with PI-88 (110).

The publication of the crystal structure of heparanase has allowed the development of small molecule drugs, the designs of which were aided by molecular modeling to inform of their likelihood of binding within the catalytic cleft. Appropriately designed small molecule drugs have the potential to exploit the characteristics of the catalytic cleft to achieve high binding affinities and favorable pharmacokinetic properties, and they may also be orally available. To date, none of these compounds have entered a clinical trial. A number of these small molecule, synthetic anti-heparanase inhibitors have been discussed in a recent publication (20) and will not be further reviewed here.

Nevertheless, of particular interest are the benzazole derivatives that have been developed using medicinal chemistry and molecular docking into the catalytic cleft to design compounds with a good fit and nanomolar anti-heparanase enzymatic activities. Two sets of derivatives have been synthesized, asymmetrical, and symmetrical derivatives (116, 117). The most recent symmetrical compounds have a relatively rigid central portion, sufficient length to span the catalytic cleft, and terminal acidic groups. Collectively these characteristics allow the compounds to fit into the cleft quite well. They occupy the same binding position as the HS tetrasaccharide that was co-crystallized with heparanase. Thus, the amino acids that interact with the HS substrate also interact with the synthetic inhibitors and the flexible, terminal acidic groups interact with the polar and basic amino acids at either end of the cleft. The best anti-heparanase of these, in terms of inhibiting heparanase enzymatic activity, is a symmetrical, thiourea glycine benzoxazole compound having an IC_50_ of 0.08 μM (117). As this compound has yet to be tested in animal models, it is impossible to say whether or not it is a good anti-cancer agent. Nevertheless, it has been shown to inhibit the invasion of a number of different tumor cell lines in an *in vitro* Matrigel assay, at concentrations that do not affect cell proliferation. Most interestingly it also inhibited the transcription and mRNA levels of the proangiogenic proteins, FGF1, FGF2, VEGF, and the metalloproteinase MMP-9 as well as heparanase itself (117). However, whether this effect was dependent upon heparanase was not determined.

## Conclusions

It is apparent from the analysis described here that heparanase is not a straight-forward anti-cancer drug target despite the wealth of evidence to indicate it contributes to tumor growth, tumor cell migration, metastasis formation, and chemoresistance. It is difficult to know how many of its contributions to cancer biology are due to its enzymatic activities, its non-enzymatic activities, or its contributions in the nucleus to the pattern of genes that are transcribed. Given this, the question is which parts of the protein should be targeted in drug development? The crystal structure revealed the catalytic cleft of heparanase is well-suited to small molecule drug development and high affinity binding by compounds like the benzazole derivatives, but whether small molecule drugs that only bind within the enzymatic cleft will inhibit the plethora of heparanase's activities *in vivo* is doubtful. Nevertheless, the *in vivo* data obtained from testing these small molecule drugs in various tumor animal models should be quite informative, and particularly so if, in these studies, analyses are also performed to reveal the expression levels of activated heparanase, the T5 heparanase variant, and Hpa2 in the tumor and its surrounding microenvironment. It is possible the *in vivo* efficacy of the anti-heparanase drugs may vary according to the expression levels of these latter two proteins, even though it is unlikely that a small molecule drug designed to specifically bind in the catalytic site will also interact with the T5 variant or Hpa2. Data obtained from *in vivo* testing of catalytic cleft specific small molecule drugs may better reveal the relative importance of the enzymatic function of heparanase, compared to its non-enzymatic activities, in tumor progression, than was the case with the anti-heparanase drugs that have currently entered clinical trial.

Clearly the catalytic cleft should be targeted, but maybe the HBD2 around residues 270–280 should also receive attention. Targeting these two areas with a “hybrid” drug comprising a small molecule like component designed to fit into the catalytic cleft, plus possibly a negatively charged saccharide-like component which would bind HBD2 outside of the cleft, may produce a drug which inhibits heparanase's enzymatic activity as well as some of its non-enzymatic activities. Moreover, binding of the negatively charged component could guide the small molecule section into the more hydrophobic catalytic cleft and so increase the kinetics of binding. However, the structure of such a negatively charged component should be informed by molecular modeling to prevent it from also interacting with the C-domain of Hpa2.

Importantly, it has been demonstrated that *in vivo* the anti-heparanase drugs will act on heparanase secreted by both the tumor and the host (20, 105), a fact that is often overlooked when assessing the *in vivo* data. Also overlooked is the fact that anti-heparanase drugs given systemically will act on the heparanase secreted by the host's tumor invading immune cells, and this could retard their immune-surveillance protective effects and so allow tumor growth. Certainly, active heparanase is of major importance for NK cell invasion of tumors and macrophage activation (53, 54).

The extent to which the issues raised in this manuscript have impeded anti-heparanase drugs from entering the clinic is unknown. Despite these issues, we believe heparanase remains a useful therapeutic target in the battle against cancer metastasis. It maybe that the clinical trials conducted to date have recruited patient populations that are too diverse in their disease, leading to an overall apparently poor response, although some patients did respond well to treatment. Whether this good response to therapy was because of the anti-heparanase activity of the drugs or their other activities is impossible to say.

## Author Contributions

DC was responsible for conceptualization, writing and project management. NG prepared the figures and provided insight into structural aspects discussed in work. DC and NG listed above have made an intellectual contribution to the work, and approved it for publication.

### Conflict of Interest

The authors declare that the research was conducted in the absence of any commercial or financial relationships that could be interpreted as a potential conflict of interest.
